# 抗肿瘤双特异性抗体研究进展与临床研发关注要点

**DOI:** 10.3779/j.issn.1009-3419.2022.101.43

**Published:** 2022-09-20

**Authors:** 媛媛 宋, 凌 唐, 琳 夏, 昕 仝, 志敏 杨

**Affiliations:** 100022 北京，国家药品监督管理局药品审评中心 Center for Drug Evaluation, National Medical Products Administration, Beijing 100022, China

**Keywords:** 双特异性抗体, 研究进展, 临床研发, Bispecific antibody, Research progress, Clinical research

## Abstract

以程序性死亡受体1及其配体1免疫检查点抑制剂为代表的药物上市，开启了抗肿瘤免疫治疗的新时代，该类药物在某些肿瘤中表现出较为突出的持续应答，如霍奇金淋巴瘤，但仍在部分肿瘤中应答率低。近年来，双特异性抗体是通过细胞融合、重组DNA、蛋白质工程等技术制备的人工抗体，可同时或先后特异性结合两个抗原或抗原表位，在肿瘤治疗中发挥协同作用，能够有效抑制肿瘤免疫逃逸，提高抗肿瘤治疗效果，成为肿瘤研发热点。本文就双特异抗体研究进展和临床研发关注要点进行综述，以期为药物研发的相关人员提供参考。

## Abstract (en)

The drugs of programmed cell death 1 and its ligand 1 immune checkpoint inhibitors have ushered in a new era of anti-tumor immunotherapy, which has shown outstanding efficacy in some tumors, such as Hodgkin lymphoma, but there is still low response rate in some kinds of tumors. In recent years, bispecific antibodies prepared by cell fusion, recombinant DNA, protein engineering and other technologies can specifically bind two antigens or epitopes at the same time or successively, play a synergistic role in tumor treatment, can effectively inhibit tumor immune escape, and improve the effect of anti-tumor treatment has become a hot spot in tumor research. This paper will summarize the clinical research and development of bispecific antibodies, to provide reference for the industry.

肿瘤免疫治疗近年来取得了较大的进展，提高了患者生存。但由于肿瘤复杂的疾病发病机制，单一靶点的免疫治疗不能有效杀死靶向细胞，接受单一抗体治疗的患者可能会产生对治疗无应答或产生抗药性。双特异性抗体（bispecific antibody, BsAb）是通过细胞融合、重组DNA、蛋白质工程等技术制备的人工抗体，可以同时或先后特异性结合两种抗原或同一抗原的两个不同表位。BsAb能够分别识别和结合两种不同的抗原或抗原表位，所以它可以把免疫细胞等效应细胞或细胞因子连接到肿瘤细胞上，进而增强对靶细胞的杀伤作用；可以结合同一肿瘤细胞上的不同抗原表位以增强其结合特异性，同时减少脱靶毒性带来的不良反应；或者结合同一免疫细胞上不同的免疫调节抗原，同时阻断/激活下游免疫信号通路，抑制或激活免疫细胞。这种具有双功能的重组抗体作为治疗肿瘤的药物理论上具有比单抗药物更高的疗效，发挥“1+1 > 2”的作用^[1]^。

双特异性抗体的概念由来已久，1960年，由Nisonoff与其合作者首次提出双特异性抗体的原始概念^[2]^。1986年，Staerz和Bevan第一次利用双特异性抗体把细胞毒性T淋巴细胞运用于癌细胞溶解实验^[3]^。之后，随着分子生物学技术的不断发展，研发者构建了多个双特异性抗体的平台。2009年欧盟批准了首个EpCAM/CD3的BsAb药物Catumaxomab用于癌性腹水^[4]^，于2017年退市。第二个BsAb药物于2014年12月美国食品药品监督管理局批准，其为安进公司研发的抗CD19/CD3双特异性抗体Blinatumomab（贝林妥欧单抗）用于复发性或难治性的费城染色体阴性（Ph-）前B细胞急性淋巴细胞白血病（acute lymphoblastic leukemia, ALL）患者的治疗^[5]^，自此正式拉开了BsAb肿瘤治疗的序幕。

然而，BsAb作为一类具有双功能的“单药”，既不同于有关的单抗，也不同于单抗的联合用药，单抗类抗肿瘤药物的临床研发思路与技术要求已经较为成熟，但是对于针对多种抗原表位的BsAb，由于结构与功能存在特殊性，是否能真正发挥“1+1 > 2”的作用，给患者带来临床获益，仍需要通过临床试验进行逐步验证。本文就BsAb研究进展和临床研发关注要点进行综述，以期为药物研发的相关人员提供参考。

## 1 双特异性抗体结构特点及作用机制

### 1.1 结构特点

单克隆抗体主要结构是由两条对称的轻链和重链组成，BsAb结构多样，通常可根据有无Fc片段进行划分，分为：不含FC片段（非IgG样BsAb）和含FC片段（IgG样BsAb）^[6-9]^：①非IgG样双特异性抗体：非IgG样BsAb通过片段化的分子设计，将多个抗原结合单元结合在没有Fc区域的分子上，从而避免了链交联问题，但是同时导致其缺乏Fc介导的相关效应功能。非IgG样BsAb主要通过抗原结合的特性发挥相应的效应机制，具有清除速度更快、半衰期较短的特点，可能在某些不良反应方面更加具有安全优势；②IgG样双特异性抗体：IgG样双特异性抗体是将两个不同靶点的抗原结合单元组合而成的IgG形态的BsAb。IgG类双特异性抗体又可以根据是否对称进一步分为对称性IgG类双抗和不对称性IgG类双抗。IgG样BsAb具有Fc片段，因此可以发挥Fc介导的效应功能，如抗体依赖性细胞介导的细胞毒作用、补体依赖的细胞毒作用和抗体依赖的细胞介导的细胞吞噬作用。有时，IgG样BsAb也可能根据功能需求，将Fc段静默处理。IgG样BsAb通过Fc片段与受体FcRn结合，血清半衰期相对更长，可能在给药频次方面更加具有优势。

### 1.2 作用机制

BsAb的作用机制为靶向两种抗原或抗原表位，可以同时阻断或激活其介导的生物学功能，或使表达两种抗原的细胞相互接近，从而增强两者间的相互作用，并以不同的作用机制介导多种特定的生物学效应^[6-9]^。

#### 1.2.1 桥联作用

BsAb可以实现细胞毒活性效应细胞的重定向功能。BsAb的一个抗原结合部位与肿瘤细胞上表达的特异性抗原结合，而另一个抗原结合部位桥联并激活效应细胞，如自然杀伤细胞、细胞毒性T淋巴细胞等。目前处于研发过程中的BsAb，主要采用细胞桥联作用，其中较为多的是T细胞桥联，作用机制为重新定向并激活表达CD3的细胞毒性T细胞，针对性杀伤表达特定抗原的肿瘤细胞，Catumaxomab和贝林妥欧单抗^[10]^采用的即为该作用机制。

#### 1.2.2 桥联受体作用

BsAb靶向同一细胞表面的两个不同受体，可以同时特异性阻断多条信号通路、蛋白或新生血管的生成，通过桥联受体可以阻断多重通道或加强信号通路从而增强抗肿瘤效果，也可以靶向介导增加肿瘤特异性和安全性。此外，BsAb还可能通过将受体A和受体B桥联在一起，激活受体下游信号通路，将两个本来不会形成二聚体的受体桥联在一起，从而产生全新的生物学信号和功能。

#### 1.2.3 介导蛋白复合物形成作用

可以用于促进蛋白复合物和膜受体蛋白复合物的形成，提高抗体药物偶联物或激动性抗体的活性。例如，2017年美国获批上市的双特异性抗体艾美赛珠单抗^[11]^是一种人源化双特异性IgG4抗体，同时桥联结合凝血因子IXa和X，从而仿真FVIII的生理功能，促进凝血酶的产生。

## 2 我国双特异性抗体药物获批及临床试验研发现状

目前，全球已有6款BsAb的单抗获批上市：Catumaxomab（EpCAM/CD3，已退市）、贝林妥欧单抗（CD19/CD3）、艾美赛珠单抗（FIXa/FX）、Amivantamab（EGFR/cMET）、Faricimab-svoa（Ang-2/VEGF-A）。其中，2018年11月，国家药品监督管理局批准药物艾美赛珠单抗用于存在凝血因子VIII抑制物的A型血友病（先天性凝血因子VIII缺乏）成人和儿童患者的常规预防性治疗以防止出血或降低出血发生的频率；于2020年12月，批准了贝林妥欧单抗用于治疗成人复发或难治性前体B细胞ALL的治疗。

值得一提的是，我国自主研发的程序性细胞死亡受体1（programmed cell death 1, PD-1）/细胞毒性T淋巴细胞相关蛋白4（cytotoxic T lymphocyte-associated antigen-4, CTLA-4）双特异性抗体卡度尼利单抗于2022年6月29日获批上市，用于既往接受过含铂化疗治疗失败的复发或转移性宫颈癌患者^[12]^。该药品的获批是基于一项关键IⅠ期临床试验，入组111例既往接受过含铂化疗失败的复发或转移性宫颈癌患者，接受本品6 mg/kg静脉输注，每2周1次，纳入全分析集的患者有99例，结果显示：经独立影像评估委员会确认的客观缓解率（objective response rate, ORR）为31.3%（95%CI: 22.4%-41.4%），完全缓解率为13.1%，缓解持续时间尚未达到，中位无进展生存时间（progression-free survival, PFS）为3.75个月，中位总生存期（overall survival, OS）尚未达到，12个月OS率为59.9%。

目前BsAb获批上市的品种个数有限，但在全球开展的BsAb抗肿瘤药物临床试验数量已经以20.44%的年增长率持续增长^[13]^，在我国BsAb药物临床试验数量也逐年递增^[11]^。大部分BsAb药物临床试验还集中在Ⅰ期和IⅠ期，少部分药物临床进入关键IIⅠ期研究。进入临床试验的研发靶点，绝大多数选用临床经过验证的靶点，在实体瘤领域以PD-1/程序性死亡配体1（programmed cell death ligand 1, PD-L1）、CTLA-4及人表皮生长因子受体2（human epidermal growth factor receptor 2, HER2）靶点为主，项目居多。在血液肿瘤领域以CD19/CD3、CD20/CD3靶点为主。目前开发的适应证主要集中在乳腺癌、胃癌、肺癌等瘤种中^[11, 13, 14]^。

## 3 临床研发关注要点

BsAb的研发遵循着药物结构决定作用机制，从而决定临床获益风险特征的规律。同时，BsAb既不同于有关单抗，也与两个单抗的联合用药有着明显的区别，以PD-L1/转化生长因子β（transforming growth factor-β, TGF-β）的双特异性抗体为例，默克公司研发的Bintrafusp alfa的双特异性抗体^[14, 15]^，其在早期探索阶段显示出了突出的疗效，但在后续的多项临床试验中，未达到主要疗效终点而终止了临床试验。因此双特异性抗体是否能真正发挥“1+1 > 2”的作用，给患者带来临床获益，仍需要通过临床试验进行逐步验证，因此在临床研发中关注以下几个方面要点。

### 3.1 确定合理的研发立题

BsAb在研发之初，应以临床价值为导向的原则，以解决临床亟待解决的问题为目标，通常情况下，BsAb的开发是在对有关单靶点/单抗的研究基础上开展的，例如改善安全性、提高有效性、克服耐药性等。在确定了拟解决的临床问题之后，开展以问题为导向的机制研究，并以此为基础有针对性地精心设计BsAb，在靶点的选择、结构的设计等方面需以临床需求指导抗体设计，从而达到研发目的。

非临床的体外和（或）体内的药效学研究结果是支持BsAb开发的重要科学依据，应重视开展相关的非临床研究，初步验证所提出的理论机制和目标，进一步支持BsAb的立题合理性。

同时，也需要注意的是BsAb既不同于有关单抗，也与两个单抗的联合用药有着明显的区别。由于BsAb结构的复杂性，使其具有更加独特的作用机制，工艺也更为复杂，也可能导致更多潜在的单药或者联合用药的安全性问题。因此，在开发BsAb时，除非通过BsAb的结构产生新的机制，否则，如果其中任一靶点具备单抗成药性时，可能需要谨慎考虑开发BsAb的必要性及合理性。例如，在某一肿瘤适应证中，AB双抗、A单抗与B单抗联合用药在该人群中取得疗效安全、安全可控，获批上市，并不代表AB双抗一定能发挥预期的协同作用；在已上市的A单抗基础上开发的AB双抗，也可能未能实现较A单抗更有临床优势的预期。

### 3.2 临床试验设计

BsAb的临床研究过程中，既要根据抗肿瘤药物临床研发的一般要求，又要注意充分发掘BsAb较单抗产品、单抗联合用药的临床优势，体现对其立题依据和初衷的印证。

#### 3.2.1 早期研究

① 关注安全性风险：BsAb的安全性风险，不完全等同于单靶点相关的安全性风险叠加，或单抗类药物联合用药的安全性风险特点。目前，双抗类药物的安全性风险主要以细胞因子释放综合征和神经毒性最为严重，同时不同双抗间还存在着差异性的毒性特征谱。因此在BsAb早期临床试验中，应该充分结合其结构特征、作用机制、靶点相关的安全性特征和非临床研究结果以及同靶点产品（如果有的话）的安全性信息等，综合对拟开发的BsAb安全性风险进行分析预判，制定临床试验期间风险管理计划，并且在临床试验中严格执行。同时科学拟定首次人体临床试验的起始剂量、剂量爬坡的幅度与速度，加入定量药理学方法，通过建模与模拟手段进行临床药理学研究；合理地定义剂量限制毒性；开展免疫原性的检测，获取临床免疫原性数据，全面评估免疫原性的影响；②最佳给药策略的探索：在设计BsAb最佳给药方案以实现获益/风险最大化时，动态表征暴露/靶点相互作用与有效性及安全性之间的关系至关重要。BsAb最佳给药策略的选择，应该是基于对药理学、毒理学、药代动力学和同靶点分子的药理学、毒理学、临床安全性、有效性数据以及暴露-响应关系（如果有）的综合评估。通过早期剂量爬坡研究可以获得药物的安全剂量范围，通常后续目标给药方案会在其安全剂量范围内进行研究和选择。由于BsAb的结构特征和作用特点具有多样性和复杂性，往往难以通过有限的剂量爬坡研究数据，仅以安全性耐受性作为单一的考量维度，来确定其最佳给药策略。因此，建议必要时，在安全剂量范围内可以选用不少于2个候选给药方案进行扩展的剂量探索研究，为确定后续研究的最佳推荐剂量和给药方案提供重要依据，鼓励研发者在临床研发期间与监管机构就剂量选择问题进行讨论；③生物标志物开发：生物标志物的运用可以寻找潜在获益人群，提高临床试验成功率，因此在早期临床试验研究过程中可加入预测和预后等生物标志物的探索研究。生物标志物的开发和应用策略应该根据双靶点或者多靶点蛋白药物的作用机制、靶点之间的生物学关系和临床意义（预测和预后价值等）进行具体设计。对于BsAb，如果只有一个靶点具有患者选择或分层意义，那么可以参考单靶点药物研发过程中生物标志物的开发和应用策略。如果两个靶点都具有患者选择或者分层意义，那么应该根据靶点之间是协同或互补的生物学关系，考虑是否需要设计组合入组条件和组合生物标志物开发策略，并且依据临床前及早期临床数据判断组合策略的必要性。由于BsAb具有独特的作用机制，且考虑到靶点之间的生物学相互作用、生物标志物的选择和使用以及具有预测/分层意义的阳性判断值的确定，可能都会与单靶点药物有所不同，因此需要依据新的临床前及早期临床研究数据重新进行确定。

#### 3.2.2 关键研究设计

BsAb的研发立题是实现有关单抗或单抗联合用药未能实现的功能，且该功能可以为患者带来有价值的临床获益。因此，在关键研究设计阶段，在采用对照研究设计时，原则上应选择最优（即反映临床实践中目标患者的最佳治疗选择）的标准治疗（standard of care, SOC）为对照。若研发立题为提高现有治疗的有效性：①相同适应证的当前最优SOC中已经包含BsAb中任一相同靶点单抗单药或者联合用药，则在随机对照研究中应选择含该单抗的标准治疗方案（单药或者联合用药）作为对照药；②拟开发的适应证标准治疗方案中不包含BsAb的任一靶点已经成药的单抗，或已有数据表明其中任一同靶点单抗单药，或者两个靶点的单抗联合治疗均无显著有效性，则无需开展与有关单抗单药或单抗联合治疗的对照“析因”研究以提供BsAb设计的合理性；在采用对照研究设计的关键临床试验中可以采用当前的最优标准治疗为对照；③如果拟开发的适应证，是对BsAb中某一相同靶点的单抗治疗耐药/难治人群，则可以选择在该单抗治疗失败人群中开展与后一线的标准治疗（或在无标准治疗的情况下，选择最佳支持治疗/安慰剂）对照的研究。

## 4 小结

生物制药技术的发展，进一步推动BsAb类药物进入高速发展阶段，尤其是在肿瘤治疗领域，BsAb药物研发呈现持续增长。此外抗体类型也已经不限于BsAb类，已有“三特异性抗体”、“四特异性抗体”等同时靶向多种抗原表位的“多特异性抗体类”药物进入临床研发阶段。

但即使技术的变革创新，仍需要关注药物研发的初心：以患者为核心，以临床价值为导向。双抗药物的研发之初的核心是解决单抗不能解决的治疗问题，为患者带来单抗治疗所不具备的临床获益。因此，在其临床研发过程中，除了遵循抗肿瘤药物一般研发规律以外，还应该注重以临床价值为导向，以结构和机制特征为基础，合理地确定研发立题，并且在后续的研发过程中，深入探索、分析和明确BsAb的临床优势，不断确证立题的合理性。
